# Rationale and design of REACT: a randomised controlled trial assessing the effectiveness of home-collection to increase chlamydia retesting and detect repeat positive tests

**DOI:** 10.1186/1471-2334-14-223

**Published:** 2014-04-24

**Authors:** Kirsty S Smith, Jane S Hocking, Marcus Chen, Christopher K Fairley, Anna McNulty, Phillip Read, Catriona S Bradshaw, Sepehr N Tabrizi, Handan Wand, Marion Saville, William Rawlinson, Suzanne M Garland, Basil Donovan, John M Kaldor, Rebecca Guy

**Affiliations:** 1 Kirby Institute, University of New South Wales, Sydney, NSW, Australia; 2 Melbourne School of Population and Global Health, University of Melbourne, Carlton, Victoria, Australia; 3 Melbourne Sexual Health Centre, Carlton, Victoria, Australia; 4 Sydney Sexual Health Centre, Sydney, NSW, Australia; 5 School of Public Health and Community Medicine, University of New South Wales, Sydney, NSW, Australia; 6 VCS Pathology, Carlton, Victoria, Australia; 7 Department of Obstetrics and Gynaecology, University of Melbourne, Parkville, Victoria, Australia; 8 Department of Microbiology, Royal Children’s Hospital, Parkville, Victoria, Australia; 9 Department of Microbiology and Infectious Diseases, Royal Women’s Hospital, Parkville, Victoria, Australia; 10 Murdoch Childrens Research Institute, Parkville, Victoria, Australia; 11 Virology Division, SEALS Microbiology, Prince of Wales Hospital, Randwick, NSW, Australia

**Keywords:** Chlamydia, Retesting, Positivity, Reinfection, Home-collection

## Abstract

**Background:**

Repeat infection with *Chlamydia trachomatis* is common and increases the risk of sequelae in women and HIV seroconversion in men who have sex with men (MSM). Despite guidelines recommending chlamydia retesting three months after treatment, retesting rates are low. We are conducting the first randomised controlled trial to assess the effectiveness of home collection combined with short message service (SMS) reminders on chlamydia retesting and reinfection rates in three risk groups.

**Methods/Design:**

The REACT (retest after *Chlamydia trachomatis*) trial involves 600 patients diagnosed with chlamydia: 200 MSM, 200 women and 200 heterosexual men recruited from two Australian sexual health clinics where SMS reminders for retesting are routine practice. Participants will be randomised to the home group (3-month SMS reminder and home-collection) or the clinic group (3-month SMS reminder to return to the clinic). Participants in the home group will be given the choice of attending the clinic if they prefer. The mailed home-collection kit includes a self-collected vaginal swab (women), UriSWAB (Copan) for urine collection (heterosexual men), and UriSWAB plus rectal swab (MSM). The primary outcome is the retest rate at 1-4 months after a chlamydia diagnosis, and the secondary outcomes are: the repeat positive test rate; the reinfection rate; the acceptability of home testing with SMS reminders; and the cost effectiveness of home testing. Sexual behaviour data collected via an online survey at 4-5 months, and genotyping of repeat infections, will be used to discriminate reinfections from treatment failures. The trial will be conducted over two years. An intention to treat analysis will be conducted.

**Discussion:**

This study will provide evidence about the effectiveness of home-collection combined with SMS reminders on chlamydia retesting, repeat infection and reinfection rates in three risk groups. The trial will determine client acceptability and cost effectiveness of this strategy.

**Trial registration:**

Australian and New Zealand Clinical Trials Registry ACTRN12611000968976.

## Background

*Chlamydia trachomatis* is the most frequently reported sexually transmitted infection in most developed countries and notification rates are increasing steadily each year [1-3]. In many countries, the greatest burden of infection is among young heterosexual men and women aged 15-24 years, with population-based prevalence estimates ranging from 3 to 6% [4-7]. High prevalence rates have also been reported in men who have sex with men (MSM), ranging from 5 to 9% for rectal infections and 3 to 6% for urethral infections [8-11].

### Repeat chlamydial infections

Repeat chlamydial infections following treatment are also common. In a prospective cohort of 16-24 year old women attending general practices in the United Kingdom, a repeat infection rate of 29.9% per year following treatment was reported (95% confidence interval [CI]: 19.7-45.4%) [12]. An Australian cohort of 1116 women aged 16-25 years reported a repeat infection rate of 18% at 3 months (95% CI: 8-34%) and 22.3% by 12 months (95% CI: 13.2, 37.6) following treatment [13]. In a review of eight studies, it was reported that 10.9% of heterosexual of men with active follow-up had a repeat infection at 4 months [14]. Higher rates of repeat infection after treatment have been reported in MSM [15]. Repeat chlamydial infections increase the risk of chlamydia-related sequelae such as pelvic inflammatory disease and infertility, when compared to initial infection [16], and in MSM, repeat rectal chlamydia or gonorrhoea infections have been associated with an increased risk of HIV seroconversion [17].

Repeat positive chlamydia tests may result from reinfection from the same partner, an infection from a new partner, inadequate treatment or treatment failure [18]. Batteiger et al. found that of the repeat positive tests among young women participating in a longitudinal cohort, 84.2% were definite, probable, or possible reinfections (different genotypes +/- unprotected sex); 13.7% were probable or possible treatment failures; and 2.2% persisted without documented treatment [18]. This study and others [19,20], have demonstrated that treatment failure with azithromycin may be a contributing factor in repeat positive chlamydia tests, and this has been the subject of recent debate [21,22].

### Chlamydia retesting

Retesting at 3 months is important to prevent onward transmission and sequelae associated with repeat infections. Given that the majority of repeat infections are the result of existing partners not being treated, repeat positivity is also an important indicator of the effectiveness of partner notification.

Although clinical guidelines in a number of countries recommend retesting after treatment for chlamydia [23-27], retesting rates are low, especially amongst men. In a recent analysis of 2008-2010 United States (US) laboratory data, chlamydia retesting rates within a year among men and non-pregnant women, were 22% and 38% respectively [28]. In a 5-year period between 2004 and 2008, the proportion of Australian sexual health service patients with chlamydia infection who were retested in 30-120 days was 8.6% in MSM, 11.9% in heterosexual males and 17.8% in heterosexual females [29]. In England, retesting rates within a year among 15 to 24 year olds in 2010 ranged from 18.4% in the National Chlamydia Screening Program dataset to 26.1% in the genitourinary medicine clinic activity dataset [30].

The true clinical impact of increased rescreening has not yet been established. Of the interventions aiming to increase retesting conducted to date, few measured repeat positive tests and none discriminated between reinfections and treatment failures [31-37]. Among the interventions which did report repeat positive test rates, despite higher retesting rates, the rate of repeat infection in all but one study, was lower in the intervention arm compared with the control arm. However only one study showed this difference was statistically different.

Based on the study by Batteiger et al. [18], the majority of repeat positive tests would be expected to be reinfections, which suggests that rescreening strategies may be reaching more asymptomatic patients and those at lower risk of reinfection. For example, in the postcard reminder study by Paneth-Pollack et al. [32], 50% of retesters in the intervention arm were asymptomatic compared to 23% in the control group. People with symptoms may be more likely to initiate retesting, and this may help to account for the lower rates of repeat infection found, despite higher retesting rates. This highlights the importance of measuring both retesting and repeat positive test rates in studies which aim to increase retesting.

The high sensitivity of nucleic acid amplification tests (NAAT) has enabled the use of self-collected specimens, such as urine and vaginal swabs, for the diagnosis and screening of chlamydia and gonococcal infections [38]. Self-collected urine, vaginal and rectal specimens have been widely used in a variety of clinical and non-clinical settings to increase access to chlamydia screening and rescreening [37,39-42] and have been found to be acceptable in men and women [43-46]. A previous study in Australia has confirmed the robustness of swabs when transported for up to a week through routine postal systems [47].

### Interventions to increase chlamydia retesting

Mailed home collection kits have been demonstrated in a meta-analysis of controlled studies by Guy and colleagues, to increase retesting rates by an average of 30% (pooled effect estimate = 1.30; 95% CI: 1.10-1.50) [48]. The same meta-analysis showed reminder strategies including phone calls (+/- letters) and postcard reminders also resulted in a modest increase in rescreening.

A reminder strategy that was not used in the studies reviewed by Guy et al. was the use of short message services (SMS) reminders. SMS and telephone reminders have been found to be effective for a variety of health related purposes such as reducing missed appointments [49] and increasing vaccination uptake [50], with SMS reminders shown to be more cost-effective [49,51]. SMS reminders have the advantage of automation, convenience, confidentiality and immediacy [52] and have been found to be acceptable in the sexual health context [53,54]. Subsequent to the review by Guy et al. [48], three Australian before-after studies have demonstrated that SMS reminders increase STI retesting rates in sexual health clinic patients [35,52,55].

### Cost effectiveness

There is limited evidence regarding the cost effectiveness of home-based versus clinic based rescreening for sexually transmitted infections. A study by Xu et al. found home-collection to be less costly than clinic-based rescreening at $54 per self-collected test versus $118 per clinic-based test [33].

We describe here a randomised controlled trial that aims to assess the effectiveness of home-collection combined with SMS reminders on chlamydia retesting rates in MSM, women and heterosexual men. The trial will also determine client acceptability and cost effectiveness of the approach.

## Methods/Design

### Study design/setting

**Figure 1:**
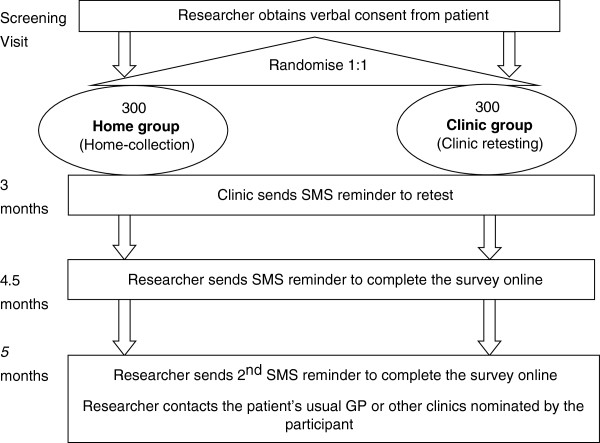
Schematic of REACT study design.

This is a non-blinded, randomised controlled trial (RCT) where individuals are randomised in a 1:1 ratio to an SMS reminder and home-based, self-collected samples (home group) or an SMS reminder and clinic testing (clinic group). The trial is being conducted in two Australian sexual health clinics (Melbourne and Sydney Sexual Health Centres). The study is diagrammatically represented in Figure 1.

### Research objectives

The primary objective of the trial is to compare the retest rate 1-4 months after treatment for chlamydia among participants in the home group (3-month SMS reminder and home-collection) compared with the clinic group (3-month SMS reminder to return to the clinic). The secondary objectives are to: compare the chlamydia repeat positive test rate in participants in the home group compared with the clinic group; assess the reinfection rate among participants who retested; determine the acceptability of home-based testing with SMS reminders; and compare health provider costs of home-based specimen collection with routine (clinic based) retesting.

### Duration of trial

The study will require 24 months to complete: 6 months for recruitment and 18 months to complete follow-up, data collection and analysis.

### Blinding

Given the nature of the intervention, it is not possible to blind the patient to their trial group. However, the statistician analysing the RCT data will be blinded to the study group.

### Ethical considerations

The REACT study protocol has been approved by the Alfred Health Human Research Ethics Committee (HREC), South Eastern Sydney and Illawarra Area Health Service HREC and the University of New South Wales HREC.

### Trial inclusion and exclusion criteria

Patients will be included if they: are aged 16 years or above; have a mobile phone; are heterosexual men (reported sexual contact only with female partner/s in the last 12 months)/MSM (reported sexual contact with male partner/s in the last 12 months)/or women; have a diagnosis of chlamydia infection (diagnosed by NAAT); and reside in a jurisdiction serviced by the sexual health clinic and plan to stay in that jurisdiction for the next six months. Patients will be excluded if they: are unwilling or unable to comply with all the requirements of the protocol; cannot speak English; are HIV positive or a current sex worker.

### Recruitment

A total of 600 participants (200 MSM, 200 women and 200 heterosexual men) diagnosed with chlamydia are required. The trial will be advertised by postcards left in the waiting room or provided to patients at the time of chlamydia testing or treatment. All positive chlamydia results will be reviewed by study nurses based at each of the clinics. Among potentially eligible patients, the nurse will contact the patient by telephone and let them know of their chlamydia diagnosis and for those not already treated, recommend they come to the clinic for treatment. During the call the nurse will give a brief overview of the study and asked the patient for permission to pass on their contact details to a member of the research team. If the patient agrees, a member of the research team will then contact the patient to explain the trial requirements and undertake a verbal consent process.

### Randomisation

Eligible patients will be randomised to the intervention or control strategies using a minimisation approach. This will maximise the balance across the risk groups (MSM, women and heterosexual men). Computer generated randomisation codes, stratified for risk group will be produced by a statistician and sealed in opaque envelopes. Once consent has been given, the research team member will select the next randomisation envelope according to the risk group of the patient and inform the patient which group they have been assigned to – the home or clinic group.

### Intervention (home testing) arm

The home-collection kit will contain the collection device, illustrated collection instructions, laboratory request form and pre-paid envelope. The collection devices will vary according to the patient’s risk group as follows: for heterosexual men the collection device will consist of a sponge-based urine collection device (UriSWAB®, Copan Diagnostics, CA, USA); for women the collection device will consist of a swab for lower vaginal self-collection and for MSM two collection devices will be provided: (i) a swab for rectal self-collection; and (ii) a UriSWAB for first-pass urine collection.

The swabs and request form will be pre-labelled with identifying information. Three months after chlamydia diagnosis, the clinic will send an SMS reminder to encourage the patient to either: (i) return to the clinic for retesting; or (ii) collect a sample/s using the collection kit and mail it to the lab with the request slip. A purpose-built database will be used to generate a list of patients due to be mailed home-collection kits. The collection kit will be mailed to the patient in an unmarked envelope by the research team. Patients will be instructed in a covering letter to collect their specimen/s and package them according to the instructions provided and mail them to the laboratory in the pre-paid envelope.

### Control (standard care) arm

Three months after chlamydia diagnosis patients will be sent an SMS reminder by the clinic to encourage them to return to the clinic for retesting. This is routine practice at the two participating clinics.

### Specimen processing, testing and results

#### Chlamydia testing

The baseline chlamydia positive specimens (600) and clinic group retesting specimens will be tested by the clinic’s usual pathology provider. Specimen collection and processing will be in accordance with the pathology provider’s usual protocol, and pathology providers will be requested to store chlamydia positive baseline and retest specimens until the end of the trial. Results will be given to patients according to the clinic’s protocol for managing test results. Treatment for chlamydia positive cases is 1g single dose azithromycin according to the routine practice [25].

All home group retesting specimens will be processed by a specialist laboratory. The laboratory will also be asked to store the specimens until the end of the trial. Results will be reported back to, and managed by the referring clinics, in the usual manner.

#### Serovar detection

Confirmation of each chlamydia serovar, and detection of genotypic variants will be determined by qPCR assay and DNA sequencing [56]. qPCR will be performed in a primary chlamydia group-specific multiplex PCR which utilises two primers and four probes, specific to all chlamydia types, including the B group (B, E, D, L1, and L2), C group (A, C, H, I, J, K, and L3) or intermediate group (F and G) serovars. The primary group-specific PCR will be used to determine which set of secondary serovar-specific PCRs to perform, as described previously [56]. When the same serovar is detected at diagnosis and follow-up, further discriminatory confirmation of relatedness using multilocus sequence typing (MLST) will be conducted [57].

### Outcomes

The primary outcome is the proportion of patients who retest between 1-4 months after a chlamydia diagnosis.

The secondary outcomes are:

1. The repeat positive test rate.

2. Reinfection rate.

3. Acceptability of home testing with SMS reminders.

4. Cost effectiveness of home testing.

### Data collection methods and variables

#### Clinical and sexual behaviour data

Among all patients who test positive at the study site in the recruitment period a range of variables (condom use, number of partners, previous chlamydia diagnoses, anal and uro-genital symptoms, treatment and chlamydia results) will be extracted from the patient management system for all episodes of care during the trial period.

#### Acceptability data

Participants in both study arms will be asked to complete a quantitative survey online. An SMS reminder will be sent to participants at 4.5 and 5 months (after ascertainment of the primary outcome). The SMS will contain the study website and the participant’s code which is linked to their patient details captured at consent. The survey will investigate participants’ living situation (with parents or not), chlamydia treatment for themselves and partners, sexual behaviour, retesting at the same clinic or elsewhere, reasons for not retesting, acceptability of SMS reminders, and for participants in the home group, the acceptability of home testing, ease of collection and retesting preferences (home or clinic retesting). On completion of the survey participants will be sent a $40 AUD voucher, irrespective of retesting. If the participant notes on the online survey that they had a chlamydia test at a clinic other than the sexual health clinic since their positive test, the participant will be mailed a consent form to obtain their permission to contact this clinic to obtain the results of this test. The consent form will then be mailed to the doctor at the clinic.

#### Cost analysis data

The cost of an organised chlamydia retesting program will be assessed from the perspective of the health care provider only. Cost data will relate to patients seen in the 12 months prior to the commencement of the study. De-identified data will be extracted from the patient management system for each patient interaction including: triage time, consultation time, staff type, risk group of client, type of clinic (regular or fast track clinical service known as the express clinic) and number of SMS reminders sent. Where these data are not available through the patient management system, (for example phone times and administration time), log sheets will be developed for the relevant staff members to document the time taken for each interaction.

### Analysis

#### Chlamydia retesting and repeat positive test rates

The final analysis of primary and secondary outcomes will be performed at the conclusion of the trial. Patient baseline characteristics will be compared by randomised group as appropriate but no formal statistical tests will be undertaken. Analyses will be conducted using STATA statistical analysis software. An intention-to-treat approach will be taken.

In the initial analysis, the percentage of individuals who returned will be cross tabulated by randomised group. The analysis will determine the effect of the intervention on primary and secondary outcomes overall and between risk groups (MSM, women and heterosexual men). The primary analysis will be retesting rates between 1-4 months after a chlamydia diagnosis. Per protocol analyses will include: i) among the home-testing arm: the percentage who retested at home compared with the clinic, and the median time to retest among those who tested at the clinic versus home; ii) in each arm: the median time to retest (overall and in those who retested positive); and iii) factors associated with repeat positivity among those who retested at 1-4 months.

#### Reinfection rate

**Figure 2:**
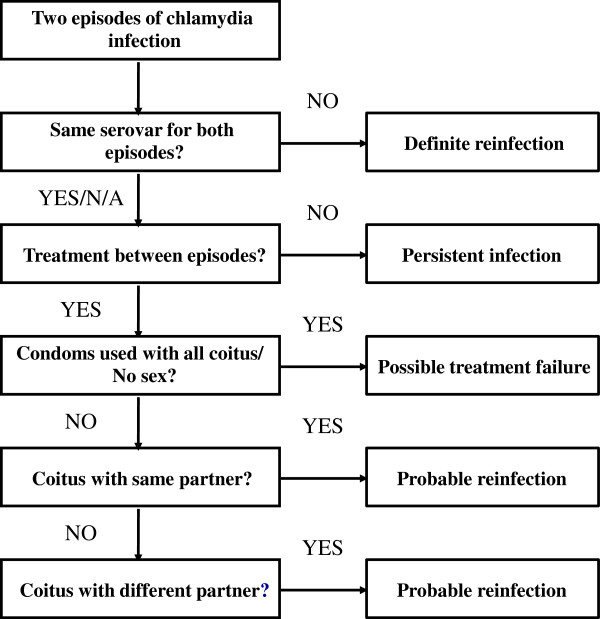
**Algorithm to differentiate between chlamydia reinfection, treatment failure and persistent infection (adapted from Batteiger et al. **[18]** and Walker et al. **[13]**).** N/A = Genotype result not available.

Consistent with the algorithm described by Batteiger [18] repeat positive cases will be discriminated using sexual behaviour data (from the questionnaires and data extracted from the patient management system) and chlamydia genotyping. To differentiate between chlamydia reinfection, treatment failure or persistent infection, we will use a modified version of the chlamydia repeat infection algorithm developed by Batteiger et al. [18] and adapted by Walker et al. [13] (Figure 2). If a participant has two infections with different genotypes, then the second infection will be considered a reinfection. If participants received appropriate treatment but had unprotected sex with their current or new partners, the second infection will also be considered a reinfection. Treatment failure will be defined as a positive chlamydia result following appropriate treatment if the participant reported either no sex between the two episodes or always using condoms with sex. An infection will be defined as persistent if the participant has two consecutive positive chlamydia test results and was not treated between episodes [18,13].

#### Acceptability

The acceptability of home-testing in the home group participants will be compared in the three different risk groups using a Chi-2 test, with breakdowns according to age group, sex, symptoms and sexual behaviour. Factors associated with acceptability will be assessed using multivariate logistic regression. The primary outcome of the analysis will be preference for home-testing.

#### Cost analysis

In order to estimate the costs of each component of retesting, a flowchart will be constructed to describe the pathway of patients from their initial chlamydia test, notification of results and treatment, to retest, either at the clinic or via home-based collection, notification of results and treatment. Labour costs will be estimated based on time, staff type and average salary. All equipment for each clinical interaction will be listed and priced according to clinic inventories. The costs of diagnostic testing will be based on the Medicare Benefits Schedule (a listing of the Medicare services subsidised by the Australian government). The cost of clinic and home-based retesting will be compared. The cost of testing at the regular clinic versus the express clinic and between risk groups will also be compared. Using the cost data and primary outcome data of retesting and repeat positive test rates, a cost-effectiveness analysis will be undertaken.

### Data storage

A central database containing de-identified quantitative trial data will be held at the Kirby Institute. All electronic files will be password protected and only the trial research staff and statistician will have access. Participants’ contact details will be securely stored separately to the quantitative trial data.

### Sample size calculation

The sample size is based on a chlamydia retesting rate of 40% in the clinic group (receiving the SMS) based on findings from previous studies. In the analysis we are interested in assessing the primary outcome in the three risk groups (200 MSM, 200 women and 200 heterosexual men) and overall. A sample size of 194 in each risk group will achieve at least 80% statistical power to detect an overall 20% difference (60% compared to 40%) in retesting between home and clinic groups. The 194 will be rounded to 200, and summed up to give an overall sample size of 600. A total sample size of 600 will achieve at least 80% statistical power to detect an overall 12% difference (52% compared to 40%) between home and clinic group for all three risk groups combined. Assuming the retesting rates above are achieved, and the repeat positivity in the clinic group is 10%, we have 80% power to detect an increase of 13% (10% compared to 23%) in repeat positivity.

### Registration

This trial is registered with the Australian and New Zealand Clinical Trials Registry: ACTRN12611000968976.

## Discussion

Chlamydia retesting at 3 months after infection is an important strategy to detect reinfections and to monitor the effectiveness of partner notification, but retesting rates are low. There is some evidence from other studies that home-based specimen collection results in a modest increase in retesting for repeat chlamydial infection as do SMS reminders, but no studies have combined these two strategies.

One of the major problems in interpreting the findings from studies to date is suboptimal study designs. Many of the studies included in the review by Guy et al. [48] were evaluated using a before-after design and it is possible that the intervention group patients may have had characteristics which facilitated rescreening irrespective of receiving the intervention. Few studies aimed to control for these differences. Some may also have underestimated retesting as a proportion of patients may have undergone screening at other health services. Several studies also had small sample sizes, precluding any analysis of the differential impact according to risk behaviour, symptoms or demographics. Another limitation of the intervention research in this area is that most studies have defined a repeat positive test as a reinfection. However repeat positive tests results may represent reinfections, persistence without treatment, or treatment failure, with varying implications for each. In addition many of the previous retesting studies included women only [31,33,36].

Most of the previous interventions which have aimed to increase retesting have focused on offering people one strategy or another. However people may choose different retesting strategies depending on their personal circumstances. For example, in a US study by Sparks et al. [58], heterosexuals aged 14 years or older were given a choice of either mailing a specimen for testing or returning to the clinic for retesting, and 30% of participants in the intervention arm chose the mailed retesting option compared with 70% who opted to attend the clinic for rescreening. Another US study of home screening by Cook et al. [31] reported that although most young women (179) received their home-collection kit in the mail, 18 (9%) opted to pick it up from the clinic, and a study in Australia found that young people were less likely to return home-collection kits if they lived with their parents [59]. These findings highlight that to maximise the effectiveness of an intervention, it is important to provide different options to suit the diverse needs and preferences of different risk groups and individuals.

This world first trial will provide evidence about the effectiveness of home collection and SMS reminders as a combined strategy, to increase retesting and detect repeat positive tests following treatment for chlamydia in three risk groups (MSM, women and heterosexual men), as well as providing information about the acceptability and cost effectiveness of this strategy. Given limited resources, offering innovative and effective ways to improve retesting rates in those at highest risk of reproductive and other chlamydia-related morbidity and HIV transmission, is an important strategy for chlamydia control.

## Competing interests

The authors declare that they have no competing interest.

## Authors’ contributions

All authors contributed to the design of the study and have read, contributed to and approved the final manuscript.

## Pre-publication history

The pre-publication history for this paper can be accessed here:

http://www.biomedcentral.com/1471-2334/14/223/prepub
